# Reimagining the status quo: How close are we to rapid sputum-free tuberculosis diagnostics for all?

**DOI:** 10.1016/j.ebiom.2022.103939

**Published:** 2022-03-23

**Authors:** Ruvandhi R. Nathavitharana, Alberto L. Garcia-Basteiro, Morten Ruhwald, Frank Cobelens, Grant Theron

**Affiliations:** a Division of Infectious Diseases, Beth Israel Deaconess Medical Center & Harvard Medical School, Boston, USA; b ISGlobal, Hospital Clínic - Universitat de Barcelona, Barcelona, Spain; c Centro de Investigação em Saude de Manhiça, Maputo, Mozambique.; d FIND, the global alliance for diagnostics, Geneva, Switzerland; e Department of Global Health and Amsterdam Institute for Global Health and Development, Amsterdam University Medical Centers, Amsterdam, Netherlands; f DSI-NRF Centre of Excellence for Biomedical Tuberculosis Research, South African Medical Research Council Centre for Tuberculosis Research, Division of Molecular Biology and Human Genetics, Faculty of Medicine and Health Sciences, Stellenbosch University, South Africa

**Keywords:** Tuberculosis, Diagnosis, Active disease, Non-sputum, Point-of-care

## Abstract

Rapid, accurate, sputum-free tests for tuberculosis (TB) triage and confirmation are urgently needed to close the widening diagnostic gap. We summarise key technologies and review programmatic, systems, and resource issues that could affect the impact of diagnostics. Mid-to-early-stage technologies like artificial intelligence-based automated digital chest X-radiography and capillary blood point-of-care assays are particularly promising. Pitfalls in the diagnostic pipeline, included a lack of community-based tools. We outline how these technologies may complement one another within the context of the TB care cascade, help overturn current paradigms (eg, reducing syndromic triage reliance, permitting subclinical TB to be diagnosed), and expand options for extra-pulmonary TB. We review challenges such as the difficulty of detecting paucibacillary TB and the limitations of current reference standards, and discuss how researchers and developers can better design and evaluate assays to optimise programmatic uptake. Finally, we outline how leveraging the urgency and innovation applied to COVID-19 is critical to improving TB patients’ diagnostic quality-of-care.

## Introduction

Tuberculosis (TB) remains a leading cause of death worldwide.^1^ TB care, which already existed in fragile and overextended healthcare systems, has been negatively impacted by the diversion of human resources and laboratory capacity for COVID-19, resulting in the number of specimens submitted for TB diagnosis plummeting from 7.1 million in 2019 to 5.8 million in 2020.^2^ 41% of the 10 million people estimated to develop TB globally each year remain undiagnosed, and TB deaths have risen for the first time in a decade.^1^ Yet COVID-19 has also demonstrated that large-scale investments (orders of magnitude higher than that seen for TB) could facilitate rapid diagnostic technology development, including the use of novel specimen types at point-of-care (POC). A similar approach for TB could yield major innovative advances.^3^

Even before COVID-19, patients evaluated for TB often experienced long delays exacerbated by circuitous pathways to care with many missed opportunities for diagnosis. The quality of TB symptom screening is often poor and primary care facilities do not often have access to tests, requiring patients to travel elsewhere.^4^ These individuals, often already attending decentralized health facilities, collectively represent an obvious first step in closing the diagnostic gap. Importantly, however, many people with TB never enter health facilities and will require identification in communities; which is often diagnostically challenging due to early-stage disease and lower pre-test probabilities, which mean tests require very high sensitivities, and specificities are a major determinant of cost efficacy. Technologies first need to demonstrate good performance and feasibility in local health facilities before they can be considered for community-based active case finding (conditions are less challenging in facilities than in communities and people with TB presenting to facilities are easier to diagnose due to increased pre-test probability). Having good performance near POC in facilities is a key criterion prior to exploring community suitability.

Closing the diagnostic gap is not just a technological challenge: it also requires ensuring that high-quality and modern tests are available^,^ in a manner that enables patients to be promptly linked to care. New technologies should be implementable at point-of-care by health care workers with minimal training, with results available in a single patient encounter.^5^ Yet, despite the advent of World Health Organization (WHO)-recommended rapid molecular tests (mWRDs) such as Xpert MTB/RIF Ultra (Ultra) (Cepheid, Sunnyvale, USA) and Truenat MTB/RIF (Molbio Diagnostics, Verna, India), much of the world still relies on sputum smear microscopy (developed in the 1880s) as the initial and often only diagnostic test^6^ despite poor sensitivity. The cost and infrastructure requirements of mWRDs remain prohibitive to scale-up and even when mWRDs are available, capacity is often not efficiently utilised or is misaligned.^7^^,^^8^ Furthermore, the use of mWRDs is typically limited to sputum-based testing. Cascade of care studies, although setting-specific, indicate that approximately 15% of patients diagnosed with TB are lost-to-follow-up and do not initiate treatment.^4^^,^^9^^,^^10^ Together, this picture shows the urgent need for decentralised testing for TB, so that the majority of patients can be tested and start treatment in one visit (ideally irrespective of their reason for initial presentation to a facility). Such tests should be sputum-free, as high-risk groups, such as people living with HIV (PLHIV)^11^ and children^12^ can often not naturally expectorate sputum and are at higher risk of extra-pulmonary TB.

Critically, there is growing recognition of the high proportion of cases identified in prevalence surveys who are a- or pre-symptomatic (presumed subclinical TB)^13^, underscoring how large numbers will be missed by current symptom screening approaches.^14^^,^^15^ Furthermore, since patients who screen positive but have early disease may not expectorate sputum, sputum confirmatory testing may not be possible. Earlier diagnosis in such people with minimal symptoms can, on an individual-level, prevent disease progression, subsequent morbidity and mortality and, on a population-level, reduce as much as 50% of transmission (importantly, community testing can enable earlier diagnoses than facility testing, resulting in even greater benefits).^16^ Moreover, as for COVID-19, such tests can also play a critical public health role if they have the capacity to identify which patients are infectious (eg, via *Mycobacterium tuberculosis* (*Mtb)* measurement in aerosol) and for how long infectiousness lasts. This reinforces the urgent need for rapid, accurate, non-invasive, and sputum-free tests for triage and confirmatory diagnosis, which have been identified by the WHO as priorities for new TB diagnostics, with the Target Product Profiles (TPP) developed in 2014.^17^

Tests and technologies that have undergone (or are undergoing) external validation, commercial development, assessment in large-scale multi-centre field evaluations, or which are judged to show high potential for TB triage, diagnosis, or the assessment of infectiousness, are prioritised for inclusion in the review. Our review is not exhaustive nor a review of biomarkers, in-house assays, centralised platforms for reference laboratories, tests to diagnose infection or drug-resistance, or the optimal way to do important community active case finding (high-quality reviews on these topics exist).^18^, ^19^, ^20^, ^21^, ^22^, ^23^ Rather, we focus on sputum-free diagnostic technologies and tests with demonstrated potential for decentralised deployment, ideally at the point-of-care at the peripheral health system level (including rural settings) to facilitate prompt clinical decision making for all forms of active TB.

## Overview of diagnostic technologies and tests by specimen type

**Figure 1:**
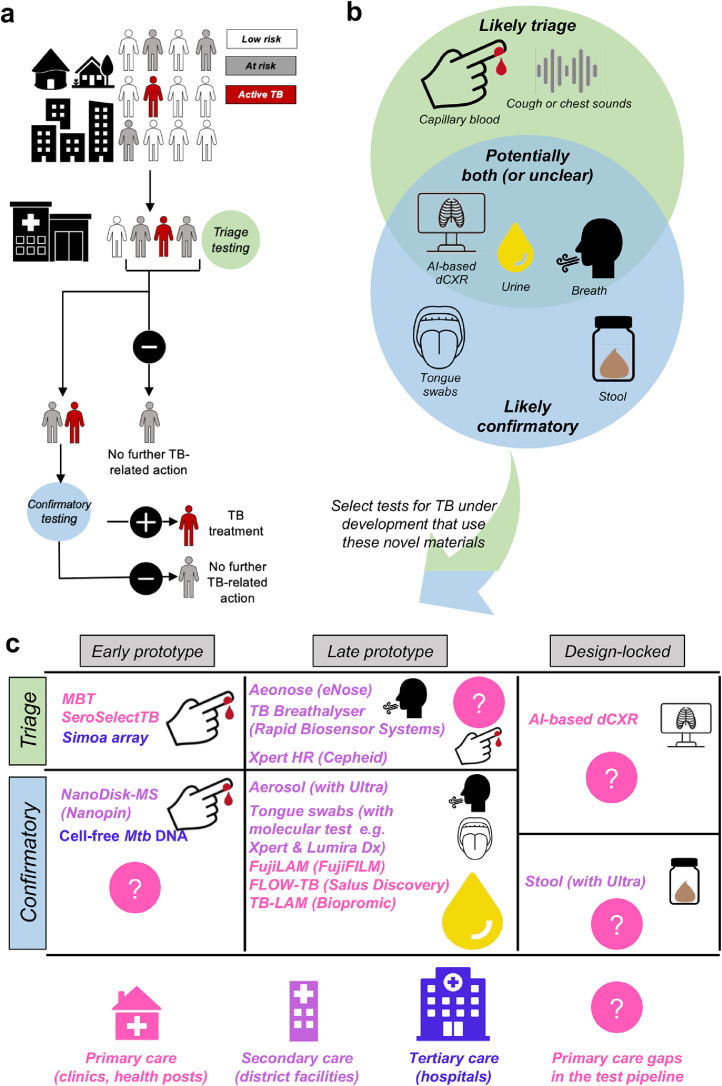
**Approaches to diagnosing TB. (a)** An overview of a typical facility-based TB diagnostic algorithm. People in a community (without risk factors for TB, white; with symptoms or risk factors for TB, grey; with TB, red) attend a health facility. After screening, all at-risk individuals are ideally identified and receive a triage test (note in some very high burden settings, all clinical attendees may be considered at risk; the definition of at-risk is setting-specific), which is done to exclude unnecessary confirmatory testing. Patients who triage positive then receive typically expensive (yet critical) confirmatory testing, which is used to inform treatment. Importantly, screening (and potentially testing) could occur in the community, however, this is not shown as most new technologies need to first demonstrate potential in facilities. **(b)** Some of the novel materials under investigation for triage or confirmation are shown (some applicable to both use cases), and **(c)** a selection of products and technologies that use these materials, their developmental stage (if known to be under commercialisation), and potential health system-level of deployment. Notably, there are insufficient late-stage and design-locked triage tests, as well as early and design-locked confirmatory tests useful for facility-based point-of-care testing (this deficit is even more serious for community-based testing, which is diagnostically and operationally more challenging). **Abbreviations:** AI: artificial intelligence, dCXR: digital chest X-ray.

Figure 1 illustrates the role of triage (which includes patients with symptoms or risk factors as well as screening of unselected populations) and confirmatory tests in a typical population of people who may have TB and move from community to clinic and hospital settings for diagnosis, the types of non-sputum specimens tested by some novel technologies under development, and a selection of these technologies, their developmental stage, likely positioning within the health system, and where gaps in the pipeline exist. Briefly, several non-invasive and easily accessible specimens hold promise as triage and confirmatory tests, however, artificial intelligence (AI)-based digital chest X-ray (dCXR) is the only design-locked triage test suitable for primary care (design-locked refers to tests where manufacturers have fixed the individual subcomponents of the assay and the core of the technology should not change).^24^ Table 1 lists various current and upcoming technologies, including their underlying mechanisms and principles, alignment with TPP criteria, commercial or in-development examples, diagnostic accuracy, pros and cons, and open questions. This table is summarized below, organised from, potentially, the least to most invasive specimens.

Tables 1a and 1b

**Table 1a:** Current and upcoming non-invasive specimen-based technologies for the rapid non-sputum-based diagnosis of TB. Technologies are listed by specimen and biomarker type (least to most invasive). Examples with best available sensitivity and specificity estimates (as well as the likely WHO target product profiles tests use case) are listed, together with known limitations. Strengths and challenges for POC deployment in high burden settings as well as open questions and considerations for researchers and implementers are discussed. Rankings of the technological level of maturity and level of confidence of available performance estimates are given.

Specimen	Biomarker	Principle and mechanism	Likely TPP for active TB	Select studies and assays≥5 years away from potential implementation, 2-5 years, ≤2 years, unclear	Level of confidence, and accuracy estimatesLow, medium, high, unclear	Strengths and challenges of technology class	Open questions and key considerations
**Breath and aerosol**	Bacterial or human metabolites or antigens	Compounds like volatile organics or *Mtb* antigens are expelled in patient aerosol and may indicate TB-associated lung inflammation and disease. Such compounds are detectable using *electronic noses*.	Triage	Aeonose (eNose)^32^	- Sufficient data exists to generate pooled sensitivity (92%) and specificity (93%) estimates^33^ for this technology class but individual products are not extensively evaluated. - Aeonose, a volatile organic compound test, has shown the most promise: in a large South African cohort of presumptive TB patients it had 90% sensitivity and 59% specificity at a rule-out threshold^98^	- Potentially implementable at scale by non-technical personnel - Biological (e.g., smoking) and environmental (e.g., pollution) factors can confound readouts^33^ - Hardware is expensive and not yet capable of providing real-time results	- Lack of independent validation data, which would be facilitated by large public data sets for discovery and training of electronic nose algorithms - Detection of some products (metabolites) must be closely timed with collection due to degradation^34^ - These technologies have diagnostic promise in people who cannot expectorate sputum, but little data exists - Could directly diagnose infectiousness, creating a new use test case^37^^,^^99^ (distinct from diagnosis) to identify potential transmission hotspots or “super-spreading” individuals for targeted case finding (pooled aerosol from a congregate setting could also be tested)
				TB Breathalyser (Rapid Biosensor Systems)^100^			
	*Mtb* DNA	Transmission occurs when *Mtb* is exhaled in aerosols, and detection could diagnose infectious TB.	Confirmatory	Face masks with filters or absorbent materials capture bacteria. This is a collection method and different assays could be applied. Blow tubes with filters are an alternate capture method.	- Promising but too early to tell - One study found a sensitivity of 87% when Ultra was applied to face masks worn by pulmonary TB cases^37^	- Sampling over long periods possible to improve yield - Specimen processing likely simpler than for sputum - Potential to detect early disease^37^ - Collection matrices require optimisation and standardisation - No performance data for assays other than Ultra like Truenat (Molbio)	
**Tongue swabs**	*Mtb* DNA	Tongue papillae filter and concentrate biomass from respiratory secretions, permitting formation of *Mtb-*containing biofilms.	Triage	Collection method and not a test itself	- Studies are few and heterogenous, no pooled performance estimates exist - Ultra applied to a single swab had a sensitivity of 88% in outpatients^41^ but only 43% sensitivity when used for active case finding in a prison^40^ - TB-LAMP^42^ applied to oral swabs had sensitivities ranging from 33-50%	- FLOQSwabs (Copan Italia) preferred^41^ - Self-swabbing, comparable to health worker-administered swabs for other pathogens^44^, appears feasible - Potential for paediatric TB^43^ - Insufficient *Mtb* may be recovered from swabs in patients with low sputum bacillary load^40^	- Performance of novel assays (e.g., next-generation LAM and NAATs unknown) may overcome sensitivity limitations associated - Optimal number of swabs, swab design, and the processing method are under evaluation and may improve the release of material from swabs -No tests purpose-built for tongue swabs yet exist
**Blood**	Host transcriptome	mRNA blood signatures associated with the immune system's response to *Mtb* have shown promise for diagnosis.^68^	Triage	Xpert Host Response (Cepheid)	- A multicentre study showed 90% sensitivity and 86% specificity^64^ -Other studies have shown lower specificities (26%^101^, 53%^102^) at >90% sensitivity	- Limited data with small numbers of cases, however, multicentre studies are emerging - Xpert HR has the most data available - RNA is labile and, for Xpert HR, time from blood collection to testing must be <30 min and stabilisation agents may be required - Cost unclear, but likely high - Potential utility treatment response monitoring, management of diseases other than TB (signature-positive patients without TB could have other infections^95^), and false-positive TB PCR results^65^, which are frequent in people with previous TB^68^^,^^84^^,^^103^	- Signatures (including Sweeney3 in Xpert HR) measured using ultra-sensitive methods (sequencing, Nanostring)^68^ struggle to meet WHO TPPs^65^^,^^66^, suggesting a performance ceiling for tests based on these signatures - Signatures insufficiently tested in PLHIV, EPTB, children, contacts, and for subclinical disease, nor head-to-head with cheap markers like CRP - Optimal placement in algorithms remains unclear
				RISK6 signature (QuantumDx)	- No product-specific data, but signature performance measured by real-time PCR is 91% sensitivity and 56% specificity (increasing to 75% in patients without previous TB)^67^		
	cfDNA	Extracellular *Mtb* DNA fragments can be detected in accessible body fluids like blood	Confirmatory	No prototypes with public data	- A systematic review and meta-analysis of different cfDNA in-house assays found a sensitivity and specificity of 78% and 97% respectively but large heterogeneity noted^62^	- Technical collection parameters influence performance^81^ - Tests on plasma appear to perform better than on other fluids^61^^,^^81^	- Still at proof-of-concept stage - Collection and detection approaches unstandardised - Product pipeline uncertain
	Host markers	Markers of inflammation made in response to disease may assist in screening and triage.	Triage	CRP (near-POC instruments like the iChroma platform (Boditech)) and other instrument-free rapid diagnostic tests are commercially-available)	- Robust meta-analysis data show sensitivity and specificity of 77% and 74% in PLHIV^104^. -At >90% sensitivity (10 mg/L threshold), specificities of 62% and 43% have been reported in HIV-negative and -positive patients, respectively^75^	- CRP outperforms WHO 4-symptom based screening in PLHIV^104^ - Instrument free CRP tests do not yet exist	- More CRP data needed in HIV-negatives and PLHIV (some studies report large specificity differences by HIV status^76^) and EPTB - Programmes lack guidance on how to implement CRP-based screening, given recent policy updates^48^ - CRP should be included as a comparator for all biomarker studies
	Antigens, cytokines, and antibodies	Altered molecular signatures are detectable in blood	Triage	MBT assay	- A LFA five marker prototype had 94% sensitivity and 96% specificity^72^	- High POC potential -Regional differences in performance may make it difficult to derive standardised panels for global use - Potential utility on saliva^105^, for progression to incident TB^106^ and treatment monitoring^107^ - Signatures should be concise (max. three targets) to improve POC feasibility	- Other than CRP, all tests are prototypes - Most assays have sensitivity limitations and require independent validation, especially in high burden, HIV-endemic settings (trials underway^108^, ^109^, ^110^, ^111^) - Stakeholders should plan how a TPP-compliant test could be implemented
				SeroSelectTB	- A LFA protype had 84% sensitivity and 97% specificity^73^		
				Simoa array panel	- 86% sensitivity and 69% specificity in a multinational global validation cohort^74^		
	*Mtb* peptides		Triage, confirmatory	NanoDisk-MS (Nanopin)	- 88% sensitivity and 96% specificity in a large Chinese cohort^79^		
	Immune cell profiling	T-cell activation markers can discriminate active disease from other forms	Confirmatory	TAM-TB (Beckman Coulter)	- A prospective study with non-TB infected controls showed 82% sensitivity and 93% specificity, which were unaffected by HIV^70^	- Demonstrated potential in children^69^ and treatment response ^71^ - Hardware (and expertise) not POC amenable and may need laboratories	- Validations required - Hardware requires simplification - Related methods that detect *Mtb* in immune cells require investigation^80^
**Stool**	*Mtb* DNA	Swallowed or disseminated *Mtb* may be detectable in stool	Confirmatory	Ultra	- A Tanzanian study in presumptive TB patients found sensitivity to range from 63-84% and specificity from 76-93% depending of the type of laboratory^45^ - A systematic review and meta-analysis of Xpert for paediatric TB found similar performance^46^	- Evidence to support use in children^46^ and sputum-scarce TB - Added complexity due to specimen processing requirements	- Data in adults (including PLHIV) scarce - Specimen provision challenging in outpatients, but rectal swabs may have utility
**Urine**	LAM	Urine is easy to collect and will have high diagnostic yield. *Mtb* cellular components (nucleic acids, complex molecules, or even intact cells) can filter through the kidney barrier. The *Mtb* cell wall and virulence factor LAM are the most promising urine markers.	Confirmatory	FujiLAM (FujiFILM)	- In a large multicentre head-to-head evaluation in HIV-negative outpatients, sensitivity was 53% and specificity 99% and highly variable between settings^57^ - Another study in HIV-positive inpatients, sensitivity was 70% and specificity 91%^56^	- Utility for EPTB^58^ - Significant technological development still needed for some assays (e.g., FLOW-TB, Mologic) compared to Determine TB-LAM (Alere) and FujiLAM - Sensitivity inversely proportion to the degree of immunosuppression (e.g., CD4 count), and this should be used to guide roll-out - For cfDNA,^61^ specialised specimen processing methods may overcome sensitivity limitations to meet TPP criteria but will detract from POC potential	- Data in important patient groups like HIV-negatives are lacking - Comparative performance data of different LAM assays is needed (in one study in HIV-negatives EclLAM, which is laboratory-based, outperformed other LAM tests that were more suitable to POC)^57^ - For urine cfDNA, many of the same considerations as for blood apply - Enrichment prior to testing may improve diagnostic yield
				FLOW-TB (Salus Discovery)	A second-generation version of the assay had a sensitivity of 86% and specificity 89% in inpatients^59^		
				TB-LAM (Biopromic)	Unclear (in development)^60^		
				TB-LAM (Mologic)	Unclear (in development)^112^		
	cfDNA			No prototypes with public data	A proof-of-concept study with an enrichment step had a sensitivity of 84% and specificity 100%^61^		

Abbreviations: LFA: lateral flow antigen, POC: point of care

**Table 1b:** Current and upcoming non-invasive non-specimen-based digital and/or AI-based technologies for the rapid non-sputum-based diagnosis of TB. Technologies are listed by specimen and biomarker type (least to most invasive). Examples with best available sensitivity and specificity estimates (as well as the likely WHO target product profiles tests use case) are listed, together with known limitations. Strengths and challenges for POC deployment in high burden settings as well open questions and considerations for researchers and implementers are discussed. Rankings of the technological level of maturity and level of confidence of available performance estimates are given.

Specimen	Biomarker	Principle and mechanism	Likely TPP for active TB	Select studies and assays≥5 years away from potential implementation, 2-5 years, ≤2 years, unclear	Level of confidence, and accuracy estimatesLow, medium, high, unclear	Strengths and challenges of technology class	Open questions and key considerations
**Audio**	Cough sounds	TB disease distorts lung architecture, affecting chest sounds in a way detectable by portable digital signal processing.^25^^,^^26^ Classification technologies are distinct from cough counting, which may have treatment monitoring utility.^113^	Triage	- Only proof-of-concept studies exist^25^ - Smartphone applications are undergoing evaluation (TimBre,^114^ Hyfe)^27^^,^^108^	- Promising but too early to tell. - One study with non-TB infected controls obtained a sensitivity of 93% and specificity of 95%.^29^	- Able to rapidly screen all facility entrants - Specimen free - Rapidly scalable on mobile phones - Abnormal sounds in the absence of TB could be used to diagnose other respiratory diseases - No external validation data	- Early-stage technology - Standardised large-scale data collection and curation required for algorithm development and testing - Performance likely highly setting-specific, especially when other lung diseases are frequent - Potential utility for evaluating post TB lung damage
	Stethoscopes			- Stethee^28^^,^^108^ and others undergoing evaluation - No published studies	- Unknown		
**Imaging**	Portable dCXR	CXR highly sensitive and increasingly feasible to be near POC due to advances in low-dose portable instruments and automated image reading.	Triage	Software products include: qXR (QURE.ai Technologies), CAD4TB (Delft Imaging Systems), and Lunit (FujiFILM), CAD4Good (EPCON) (see AI4HLTH for a complete overview)^30^	- In a very large evaluation in Bangladesh, qXR and CAD4-TB have the highest specificities (both 73%) at >90% sensitivity^50^	- Automated reading systems can overcome radiologist shortages and are WHO-endorsed^48^ - Expensive equipment and infrastructure (including high-speed internet) required^52^, however, existing equipment may be retrofitted such that analogue images can be converted to digital ones - Market rapidly evolving and selection of the right solutions challenging	- Challenges in identifying which settings for prioritisation, including case finding scenarios where pre-symptomatic TB can be detected^115^ - Achieving adequate throughput to maximise utility is challenging - Unclear how these technologies will benefit diagnosis of other diseases - Open source social impact programs such as CAD4Good may reduce costs^30^
	POCUS	Improving affordability and portability of ultrasound devices has led to interest in the use of POCUS.	Triage	Many (most standard point-of-care ultrasound machines can be used)	- Systematic review reported sensitivities ranging from 73-100% for subpleural nodules detected and 47-80% for lung consolidation^54^	Potential to expand diagnosis of extrapulmonary TB and increase diagnostic yield in populations such as children or PLHIV	- Limited data, currently majority of studies in adults and for pulmonary TB - Effects of operator and machine variability unclear - Optimal imaging protocols unclear

### Audio

The growing interest in AI has opened the possibility of non-invasively detecting cough changes or lung sounds that differentiate people with and without TB, with potential for use as a triage test.^25^^,^^26^ This could be done using portable digital recording and signal processing mobile phone enabled applications^27^ or digital stethoscopes.^28^ Such *specimen free* technologies, could provide a more objective measure of symptoms such as cough, in contrast to subjective and challenging syndromic screening, and are potentially scalable given a theoretical ease-of-use and rapidity of turnaround (seconds). Nonetheless, these technologies are early stage, with signatures yet to be externally validated and limited clinical data to support use (one study reported a sensitivity of 93% and specificity of 95% of AI-based cough classification when comparing the coughs of people with and without TB).^25^^,^^29^ Initiatives to assemble large collections of cough audio recordings that can be used to train and test algorithms are needed, similar to what was successfully done for dCXR, where a global image archive from diverse settings and populations was made available to developers.^30^ Performance of audio analyses is likely to be setting-specific and impacted by the prevalence of other lung diseases in the population but, as a new field, *acoustic epidemiology*^31^ holds tremendous promise for identifying patients for confirmatory testing, monitoring treatment response, and assessing population-level lung health.

### Compounds in breath

Differences in signatures of volatile organic compounds expelled in breath can be analysed to facilitate detection of pulmonary diseases like TB, most likely as a triage test. Such compounds can be detected using electronic nose devices or captured and concentrated using a collection bag and detected by methods like gas chromatography.^32^ Although a recent systematic review suggested that electronic nose diagnostic tests may have high accuracy (pooled sensitivity and specificity both 93%),^33^ these tests were often evaluated in case-controlled studies comparing people with TB to healthy controls and not in the intended setting of use (potential spectrum bias leading to accuracy overestimation). Other tests in this review had sensitivities from 62-100% and specificities from 11-84%.^33^ Notably, however, data were insufficient to obtain pooled estimates for a single type of test. While the non-invasive nature of such tests is appealing, large public datasets are, like for audio, needed for algorithm generation and training. However, methods of collection (which can lead to the combination of volatile organic compounds with sample bag material), analytical approaches, or confounders (like pulmonary comorbidities or smoking), can all introduce variation. Important operational challenges, including the need for prompt (ideally real-time) volatile organic compound detection post-collection (samples undergo degradation after collection) and hardware complexity remain.^34^ Tests that require specimen shipment off to a central laboratory hold less appeal.

### *Mtb* in aerosol

Tests to diagnose subclinical (asymptomatic) TB are lacking. This state (bacteriologically-positive TB in those reporting no symptoms and typically not seeking healthcare) is more prevalent than previously estimated, with highly variable duration (six months to beyond five years)^35^^,^^36^ and may be responsible for more than half of TB transmission.^16^ The detection of *Mtb* bacilli or DNA in aerosols has been facilitated by the development of face masks, with capture filters or absorbent materials, or blow tubes with a capture filter.^37^, ^38^, ^39^ Although capture methods are still under development, early results have been promising, suggesting high diagnostic yield (one study demonstrated 87% sensitivity when Ultra was used to analyse samples collected from face masks), and may identify patients with subclinical disease earlier than sputum-based sampling.^37^ Furthermore, these methods can be applied to people before they cough: sampling aerosol from tidal breathing has yielded culturable *Mtb* bacilli, suggesting cough is not a transmission prerequisite.^39^ Detection of *Mtb* in expelled aerosols may thus play a role in identifying early disease, thought to be important for transmission,^41^ and assessment of treatment response (important for infection control); however, this technology remains early stage.

### Tongue swabs

*Mtb* in tongue papillae biofilms may be detected using existing molecular technologies such as Ultra^40^ with high sensitivity: 88% relative to sputum Ultra^41^ based on single flocked swab sampling in symptomatic outpatients; however, sensitivity may be heavily setting dependent (a prison-based active case finding study reported a sensitivity of Ultra on a single swab of 43%).^40^ Tongue swab-based diagnoses are also possible using TB-LAMP.^42^ In general, however, the optimal number of swabs and approach needed to maximize DNA recovery during processing remains the subject of active investigation. Next-generation ultra-sensitive tests may further increase the feasibility of tongue swab-based methods as current generation tests still have sensitivity limitations and automated extraction and wash steps were designed for sputum not tongue swabs. If increased sensitivity could be achieved, tongue swab-based approaches could be particularly useful as a diagnostic test in children, where feasibility with Ultra from tongue swab has been demonstrated^43^, as well as other sputum-scarce populations. Lastly, tongue swab (self)-collection has been done reliably by patients for SARS-CoV-2 testing^44^, which is an important consideration for scale-up and potential community- or home-based TB testing in under-resourced settings.

### Stool

Tests on stool currently have the most utility in diagnosing childhood TB, given the challenges in obtaining sputum; although their suboptimal sensitivity (pooled sensitivity from nine studies was 67%)^45^^,^^46^ remains a barrier. In adults, stool-based tests may play a role in the diagnosis of extrapulmonary TB, particularly in groups such as PLHIV who are more likely to have disseminated disease. Rectal swabs may facilitate specimen collection and processing, making the approach more amenable to POC during a single encounter, although accuracy data are limited (one study demonstrated the sensitivity of stool Xpert testing using FLOQswabs was 47%).^47^ Generally, stool-based approaches are hampered by the need for non-POC specimen processing.

### Imaging

Several AI computer-aided detection (CAD) software platforms for TB triage^30^ are recommended by WHO as an alternative to human reading, given their potential to overcome the lack of qualified readers and the overwhelming need for improved triage methods given limited resources for confirmatory testing.^48^ An evaluation of 1032 images demonstrated that six out of 12 CAD platforms (Qure.ai, DeepTek, Delft Imaging, JF Healthcare, OXIPIT, Lunit) performed similarly to an expert reader, only three of which (Qure.ai, Delft Imaging and Lunit) performed significantly better than an intermediate reader.^49^ A large evaluation of almost 24 000 outpatients, the majority of whom had symptoms, demonstrated that all five of the algorithms evaluated reduced the number of molecular tests required by 50% while maintaining an overall sensitivity of 90%. Two products: qXR (qure.ai, India) and CAD4TB (Delft Imaging Systems, Netherlands) met the triage TPP criteria.^50^ Ongoing challenges include the need to adapt score thresholds to local epidemiology and use case scenarios (e.g., triage versus mass population screening, different clinical settings, pre-symptomatic patients), as software performance, particularly with respect to specificity, can be variable and precludes it from serving as a diagnostic test.^51^ While the equipment cost remains prohibitive^52^, there are advances in the use of POC imaging devices (including smartphones) that may bridge this barrier as the market rapidly evolves, particularly given the potential of this technology class to be used as part of integrated management of other diseases such as COVID-19, which may ensure adequate throughput to justify costs.^53^ Other imaging technologies of interest include point-of-care ultrasound (POCUS), for which a systematic review revealed sensitivities from 73-100% for subpleural nodules and 47-80% for lung consolidation; however, data are limited with high risk of bias.^54^

### Urine

Urine is appealing given ease-of-collection, limited infection control requirements, and potential for extrapulmonary and pulmonary TB. *Mtb* cellular components (nucleic acids, molecules, cells) can filter through the kidney barrier into urine. Urine lipoarabinomannan (LAM) is the only WHO-recommended biomarker for TB diagnosis (specifically the AlereLAM test).^55^ Other next-generation LAM assays from FujiFilm, SD Biosensor, Biopromic, Salus, and others are at different developmental stages. Studies comparing SILVAMP TB LAM (FujiFilm) to AlereLAM demonstrate a higher sensitivity (70% vs. 42% in PLHIV, 67% vs. 53% in people without HIV).^56^, ^57^, ^58^ Several ultra-sensitive 3^rd^ generation LAM assays^59^^,^^60^ to be used irrespective of HIV status will enter trials in 2023. Lastly, emerging data points to a potential role for urine cell-free DNA for TB diagnosis;^61^ however, the need for specialised processing methods must be overcome for wide use.

### Blood

While there are no validated blood tests for active TB, there is increasing optimism regarding the detection of antigens, immune cell profiling, host transcriptomics, or cell-free *Mtb* DNA.^62^ Several studies have demonstrated associations between host mRNA signatures and TB risk, although preventive therapy guided by one such mRNA biosignature failed to reduce TB incidence in a large randomised controlled trial.^63^ Despite commercial progress to develop mRNA biosignature assays, foremost of which is the Xpert Host Response (Xpert HR) cartridge that detects a 3-gene signature in capillary blood directly using the widely-deployed GeneXpert platform,^64^ independent validations^65^^,^^66^ demonstrate most transcriptomic biomarkers will not meet the WHO diagnostic accuracy criteria for triage or confirmation.^65^, ^66^, ^67^ Furthermore, tests will still likely require costly processing methods that preclude scale-up.^68^ Tests that measure T-cell activation such as TAM-TB have demonstrated potential for active TB in children (83% sensitivity vs. culture)^69^^,^^70^ and may have a role in treatment response monitoring,^71^ however, TAM-TB's reliance on flow cytometry may restrict deployment to clinical laboratories. Detection of TB antigens^72^, ^73^, ^74^ and other molecular biomarkers also holds great potential for scale-up in POC tests. For example, despite being a non-specific biomarker of inflammation, C-reactive protein (CRP) is recommended by the WHO as a screening test in PLHIV given its superior accuracy for TB compared to symptom screening^75^^,^^76^; however improved implementation guidance is needed.^77^ Other assays to detect inflammatory biomarkers^78^ or TB peptides^79^ remain in the prototype phase but have POC deployment potential. Proof-of-concept data has been generated for *Mtb* DNA detection in peripheral blood mononuclear cells^80^ and cell-free DNA but no prototypes with public data are available.^62^^,^^81^

### Outstanding questions: considerations for test developers and evaluators

Select considerations for developers and evaluators are in Table 2, together with issues for implementers and policymakers, which are interlinked and fall across multiple themes, are discussed in the following sections.

**Table 2:** Key challenges that need to be overcome for new diagnostic technologies to have a transformative impact on the TB epidemic. These issues are complex, multifactorial, and interdisciplinary, however, together they create a significant barrier to the adoption of promising new non-invasive TB tests at the point-of-care. When developing and planning to implement a new test, all factors require consideration, otherwise potential impact is undermined.

Colour theme key: Red- technical; orange- programmatic; purple- policy.

Abbreviations: POC: point of care, DST: drug susceptibility testing.

From a technical perspective, developing POC tests is challenging due to disease complexity and the slow growth of *Mtb*. While ultra-sensitive tests are still needed to detect early disease (especially in the context of active case finding),^36^^,^^82^ as tests slowly increase in sensitivity, diagnostic accuracy assessments may be compromised by reference standard limitations, especially as bacteriological tests like liquid culture are often only applied to sputum.^83^ Thus, positive results from highly sensitive tests could be classified as false positives, representing potentially missed treatment opportunities. This false-positivity issue may be of less concern for tests that use antigens or DNA rather than host biosignatures such as mRNA, however, challenges remain: for example, sputum Ultra trace semi-quantitation results are often culture-negative, especially in settings where patients have high rates of previous TB or a where there is a high background intensity of TB transmissiont.^84^ Biomarkers like host RNA and CRP have potential for resolving Ultra false-positive results (if biomarker levels are low),^65^^,^^75^ thus Ultra traces could, for example, be reflexed to a test like Xpert HR. Aside from limit of detection differences, other reasons for discrepant results include variations in specimen quality and processing, extra-pulmonary TB, and non-culturable *Mtb* (the clinical relevance of which is not well understood). Composite reference standards that incorporate additional information (e.g., imaging and treatment response) may be useful, however, these substantially increase expense. Extra-pulmonary TB, which accounts for around 16% of all global TB cases,^1^ presents additional diagnostic challenges due to invasive sampling to obtain site-specific tissue or fluid, on which the sensitivity of existing tests is generally lower than on pulmonary specimens.^85^ In addition, for several tests there are data or potential for extra-pulmonary TB, indicating that invasive sampling could be obviated (see Table 1).

Although rapid POC biosignature tests may contribute substantially to reducing those patients lost to follow up between diagnosis and treatment, one limitation (especially for host biomarker tests) is that they are unlikely to provide drug susceptibility information to guide appropriate treatment, in contrast to tests that directly detect *Mtb*. The latter should be given higher priority by developers and implementers in high DR-TB burden settings.

Consideration of the feasibility of obtaining alternative specimen types (such as urine or stool) in routine settings, although non-invasive, is important to avoid undermining potential benefits. Blood volumes for biomarker tests could also be a concern and these should ideally require only a finger prick. During development, any platform-based assay should consider potential maintenance and quality assurance needs for field use and address these during validation or early field evaluation studies, rather than leaving this for post-market evaluation. Furthermore, local communities’ perceptions about specific specimen types (e.g., stool) or tests (e.g., audio) should be carefully studied. TB diagnostics research should utilise interdisciplinary quantitative and qualitative approaches to maximise the high quality uptake of testing algorithms by patients and health care providers.

### Outstanding questions: Key issues beyond test performance that need to be addressed by programmes and policy makers for implementation and scale-up

Systematic reviews evaluating the effect of Xpert MTB/RIF and LAM on patient-important outcomes like mortality demonstrate the challenges of moving from diagnostic test accuracy assessment to proving impact.^53^^,^^86^ Careful evaluation of intended and actual test placement and use within the wider TB care cascade is needed to understand potential implementation barriers,^87^^,^^88^ such as gaps in linkage and engagement with care. The global roll-out of Xpert MTB/RIF has provided key lessons for policy makers and program implementers,^89^ which include recognising that high costs and unfulfilled quality assurance and maintenance needs in chronically underfunded programmes will continue to result in underutilisation.^90^ Technologies can mitigate these barriers if a POC test can be electricity-free, deployable at wide temperature ranges, have long shelf life, and the potential for online and offline use. National TB programmes must facilitate less restrictive use of new tests to increase utilisation volumes, requiring price negotiations with test manufacturers, and comprehensive health worker training. Furthermore, continuous quality assurance programmes should ensure tests are being used, interpreted, and acted upon correctly and promptly.^91^

Improving TB diagnosis, particularly given the renewed focus on active case finding, needs to be viewed within the broader context of health systems strengthening. Despite the release of the WHO's first Essential Diagnostics List in 2019,^92^ country-level investment, particularly to strengthen primary health care in high TB incidence settings, falls short of ensuring availability of essential tests and infrastructure, such as dCXR with CAD in peripheral healthcare settings.^93^ Syndromic rather than test-guided management remains highly prevalent.^94^ It is critical that health care providers are trained to act rapidly on test results to link patients to care, as well as to interpret negative tests to determine appropriate follow-up investigations and avoid patients being lost to care without a working diagnosis.

Even the most promising triage tests (CRP, host transcriptome signatures) have specificity limitations, since other conditions (viral infections, cancers) can produce readouts that may resemble TB,^95^ which impact implementation potential given that triage test specificity is the primary cost driver.^96^ Hence, with the introduction of novel tests, a negative test in a triage-positive person should inform further non-TB diagnostic decision making to justify these tools' cost. Most modelling exercises do not factor in potential benefits of non-TB diagnoses, which may underestimate overall impact. These diagnostic algorithms, including alternative diagnoses to be considered in patients without TB would need to be setting- and population-specific.

The COVID-19 pandemic has prompted the international scientific community to develop highly accurate SARS-CoV-2 diagnostic tests in record time, with advances in sample collection, including specimens such as saliva, oral swabs and absorbent strips in facemasks, and decentralised testing, including the use of home-based testing kits.^14^ The TB diagnostic community needs to follow similar development pathways, including integrated testing for respiratory diseases such as TB and COVID-19, utilizing multi-disease molecular diagnostic platforms (for example, GeneXpert and Truenat), which leverages the setting-specific feasibility of automatically testing a specimen for multiple diseases. Digital connectivity tools can facilitate community-based testing, referrals, contact tracing, linkage to care and/or indicate the need for alternative clinical evaluation.^3^^,^^97^

## Conclusions

Most upcoming technologies for sputum-free TB tests are at an early developmental phase. Furthermore, many methods that use novel specimens are still reliant on existing molecular tests not originally designed for that purpose (e.g., Ultra on tongue swabs) or require infrastructure unavailable at primary care. Despite serious gaps in the diagnostic pipeline, exploring the potential for several prototype tests that show promise for POC deployment across different use case scenarios (TPPs) is critical. Nonetheless, given growing recognition of the high proportion of people with subclinical TB and the nuances of use case scenarios for screening versus triage, revised TPPs are needed. Key needs to accelarate test development include publicly available standardised datasets and large-scale validation cohorts that facilitate test design and evaluation from the outset in populations and settings that reflect their final intended field of use. To overcome these barriers, it is critical to leverage the resources and diagnostic innovation and momentum behind the COVID-19 response, including scale-up of SARS-CoV-2 test capacity and connectivity tools. Only then can the promise of novel sputum-free TB tests be realised.

### Search strategy and selection criteria

Data for this Review were identified by searches of the PubMed database, and references from relevant articles, using the search terms (Tuberculosis or TB) AND (diagnosis) without restrictions based on language, date, study type, or setting. Given the focus on novel technology, additional references were obtained from abstracts, meeting reports, and clinical trial registration websites, where relevant.

## Contributors

RRN, ALGB, and GT drafted the initial manuscript with critical input from MR and FC. All authors read and approved the final version of the manuscript.

## Declaration of interests

M. Ruhwald reports working for FIND. FIND conducts multiple clinical research projects to evaluate new diagnostic tests against published target product profiles that have been defined through consensus processes. These include studies of diagnostic products developed by private sector companies who provide access to know-how, equipment/reagents, and may contribute through unrestricted donations according to FIND policies and in line with guidance from the organisation's external scientific advisory council. FIND does not attribute any financial value to such access. The other authors have no competing interests to declare.
